# All‐Printed Flexible Hygro‐Thermoelectric Paper Generator

**DOI:** 10.1002/advs.202206483

**Published:** 2023-01-22

**Authors:** Haoyu Shen, Ke Xu, Yulong Duan, Peilin Wu, Zhiyun Qian, Yonghao Chen, Yao Luo, Chaocheng Liu, Yang Li, Jiedong Cui, Detao Liu

**Affiliations:** ^1^ School of Light Industry and Engineering State Key Laboratory of Pulp and Paper Engineering South China University of Technology Wushan Rd., 381#, Tianhe District Guangzhou Guangdong 510640 China

**Keywords:** hygrothermal resources, hygro‐thermoelectricity, ion‐electron ink, paper generator, sustainability

## Abstract

The conversion of ubiquitous hygrothermal resources into renewable energy offers significant potential for cable‐free, self‐powered systems that can operate worldwide without regard to climatic or geographic limitations. Here, an all‐printed flexible hygro‐thermoelectric paper generator is demonstrated that uses bifunctional mobile ions and electrons to make the moist‐diffusion effect, the Soret effect, and the Seebeck effect work synergistically. In the ordinary hygrothermal settings, it generates an unconventional hygro‐thermoelectric output pattern and shows almost a dozen‐fold increase in positive hygro‐thermopower of 26.70 mV K^−1^ and also another negative hygro‐thermopower of −15.71 mV K^−1^ compared to pure thermopower. A single paper generator can produce a giant 680 mV displaying typical cyclic sinusoidal waveform characters with volt‐sized amplitudes. The ion‐electron conductive ink is easily printable and consists primarily of a Bi_2_Te_3_/PEDOT:PSS thermoelectric matrix modulated with a hygroscopic glycerol that releases ion charges for moist‐diffusion effect and Soret effect, as well as electron charges for Seebeck effect. The emerged hygro‐thermoelectric harvesting strategy from surrounding hygrothermal resources offers a revolutionary approach to the next generation of hybrid energy with cost‐efficiency, flexibility, and sustainability, and also enables large‐scale roll‐to‐roll production.

## Graphical abstract

A conversion of ubiquitous hygrothermal resources into renewable energy offers significant potentials for cable‐free, self‐powered systems that operates globally without climate or geography limitations. The all‐printed hygro‐thermoelectric paper generator is reported to make moist‐diffusion effect, Soret effect, and Seebeck effect work synergistically for harvesting giant power, proposing a revolutionary approach to collect useful renewable energy from ambient hygrothermal resources.

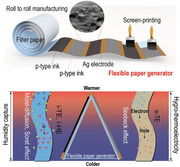

## 1 Introduction

In contrast to the renewable energies such as solar, wind, and hydrogen, which are currently constrained by either geographical or climatic requirements, the alternative promises hygroelectricity (HE)^[^ ^1^, ^2^, ^3^, ^4^, ^5^, ^6^ ^]^ or thermoelectricity (TE),^[^ ^7^, ^8^, ^9^, ^10^, ^11^, ^12^ ^]^ which rely on the low‐grade humidity or heat resources in ambient air environments, that is highly desirable due to its affordability, ubiquity, and easy accessibility without geographic or climatic limitations, thereby enabling the useful generation of renewable energy to support wireless, self‐powered systems.^[^ ^13^, ^14^ ^]^ However, in recent years the most important research has generally focused on two distinctly different energy harvesting strategies of HE and TE; HE is typically produced by the moist‐diffusion effect,^[^ ^15^, ^16^ ^]^ while TE is generally induced by the Seebeck effect, the Soret effect, or the thermogalvanic effect,^[^ ^17^, ^18^ ^]^ and more importantly, each of them operates completely the different settings.

The figure of merit (ZT), ZT = *S* ^2^ *Tσ*/*κ* ^[^ ^19^ ^]^ (Seebeck coefficient *S*, electrical conductivity *σ*, and thermal conductivity *κ*), is widely used to assess the superiority of thermoelectric materials for thermopower. Here, the promising thermoelectric materials are suggested to have higher electrical conductivity and Seebeck coefficient, but lower thermal conductivity.^[^ ^20^ ^]^ Concerning this criterion, the Seebeck effect is induced by the directionally mobile electrons or holes as energy carries from hot side to cold side under temperature gradient (*T*_g_),^[^ ^21^ ^]^ typically employing narrow‐bandgap inorganic (e.g., Bi_2_Te_3_)^[^ ^22^, ^23^ ^]^ or/and organic (e.g., PEDOT:PSS and polyaniline)^[^ ^24^, ^25^ ^]^ semiconductors and also their hybrid composites^[^ ^26^ ^]^ as electronic thermoelectric (e‐TE) materials. In particular, the widely used Bi_2_Te_3_ semiconductors and their alloyed e‐TE materials have attracted considerable attention due to their satisfactory electronic conductivity and Seebeck effect; however, suffering from its naturally high thermal conductivity as well as the resultant lower ZT value, these e‐TE materials face more serious thermoelectric blockages.^[^ ^27^, ^28^ ^]^ The thermal conductivity of Bi_2_Te_3_ typically reaches up to 1.0 W m^−1^ K^−1^,^[^ ^29^ ^]^ showing that it is against the improvement of ZT value. Especially, when employing them in a flexible configuration, their inherent brittleness has negatively affected both their native performances and long‐term stability. Because of this, the flexible Bi_2_Te_3_ materials with high ZT properties are highly coveted yet still challenging to achieve.^[^ ^30^ ^]^ Fortunately, it has been recently reported that combining the organic e‐TE materials of such PEDOT:PSS with much lower intrinsic thermal conductivity with inorganic Bi_2_Te_3_ semiconductors to synthesize organic–inorganic thermoelectric composites seems to show better ZT and flexibility.^[^ ^31^, ^32^ ^]^ Notably, the electronically conductive polymers nevertheless have outstanding electrical conductivity and thermopower while maintaining intrinsic thermal conductivity ranging from 0.08 to 0.60 W m^−1^ K^−1^.^[^ ^33^ ^]^

Unlike electron or hole charges as energy carriers in e‐TE materials, ionic charges in ionic thermoelectric (i‐TE) materials can generate different types of entropy through ionic thermo‐diffusion effect (known as the Soret effect). It is pointed out that the Soret effect is induced by the directional migration of ion charges from the hot side to the cold side, potentially producing enormous i‐TE power that is tens or even hundreds of times higher than those of the Seebeck effect. This power generally ranges from a few mV K^−1^ to 30 mV K^−1^.^[^ ^34^ ^]^ For example, the flexible quasi‐solid state ionic gels of ionic liquids and poly(vinylidene fluoride‐*co*‐hexafluoropropylene) showed the highest Seebeck coefficient ever reported by scientific communities, up to 26.1 mV K^−1^.^[^ ^35^ ^]^ This superiority offers a brand‐new potential opportunity toward using low‐grade heat to efficiently power a far broader scope of electric devices. Whereas, when subjected to a temperature gradient, the operating mechanism of ions‐induced thermopower by Soret effect uncovers intrinsically homologous to moist‐induced HE working with directionally mobile ionic charges released by moist‐induced effect under ambient humidity.^[^ ^36^ ^]^ Ionic conductors of such i‐TE and i‐HE materials only employ ion charges as energy carriers to generate potential differences across the electrical double layer (DEL) structure while e‐TE uses electrons or holes as energy carries. However, a fact has been currently ignored that in most cases the natural waste heat resource coexists with the ubiquitous gaseous moisture in real‐world surroundings, making it extremely challengeable, that makes either i‐TE or i‐HE to work independently.^[^ ^37^, ^38^, ^39^, ^40^, ^41^ ^]^ As a result, the HE induced by moist‐diffusion effect interacts theoretically with i‐TE production, which probably impacts the electron charge migrations induced by Seebeck effect.

Because of their fundamentally different physical mechanisms of action, it still remains unclear whether moist‐diffusion effect, Soret effect, and Seebeck effect can work synergistically to boost the final power generation in a single generator system. The concept of “hygro‐thermoelectricity” (HTE) is defined herein as the energy generated by enabling the moist‐diffusion effect, the Soret effect, and the Seebeck effect to work synergistically in hygrothermal surroundings. Unfortunately, the currently reported strategies only focus on the independent harvesting of hygroelectricity or thermoelectricity, neglecting the giant advantages of their possible synergistic effects^[^ ^42^, ^43^, ^44^, ^45^, ^46^, ^47^ ^]^ when they operate in intrinsically different working principles.

In this work, we report a novel HTE harvesting strategy that combines the moist‐diffusion effect, the Soret effect, and the Seebeck effect to work synergistically using fully printable flexible paper generator in hygrothermal environments. The emerging technology herein is to fully print the synthetic ion‐electron conductive ink and Ag electrodes directly onto the commercial filter paper substrates to make a flexible paper generator. It has a unique ion‐electron transport layer structure modulated by a highly hygroscopic glycerol, which effectively makes the moist‐diffusion effect, the Soret effect, and the Seebeck effect work together. The organic PEDOT:PSS and the inorganic Bi_2_Te_3_ and the carbon nanotube (CNTs) are integrated into a bifunctional ion‐electron conductor that employs the H^+^ charges ionized from the regenerated PSS as ionic energy suppliers contributing to the moist‐diffusion and Soret effect; and it also uses the electron or hole charges derived from the Bi_2_Te_3_, PEDOT semiconductors as electronic energy suppliers contributing the Seebeck effect. Here, the synergistic work of directional ion and electron charge migration within the conductive ink layer efficiently converts the surrounding hygrothermal resources into a giant final HTE generation.

## 2 Results and Discussion

**Figure 1:**
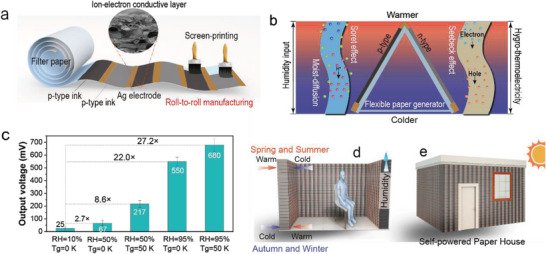
a) Schematic diagram of roll‐to‐roll manufacturing of all‐printed flexible hygro‐thermoelectric paper generator. The n‐ and p‐type ion‐electron conductive ink and Ag electrode are alternately screen‐printed onto the surface of filter paper substrate with partially embedded structure. b) Schematic figure of ion and electron charges as energy carries for hygro‐thermoelectricity generation in flexible paper generator by making moist‐diffusion effect, Soret effect, and Seebeck effect work synergistically under the hygrothermal resources. c) Comparative voltage outputs were investigated by employing various ambient humidity (RH = 10%, 50%, and 90%) and temperature gradient (Tg = 0 and 50 K). d) The virtual application scenarios of perennial hygro‐thermoelectricity production under the continuous generation of temperature gradient between indoors and outdoors when people control comfortable indoors temperature using air conditioners during spring, summer, autumn, and winter seasons; and more importantly, regular humidity is mostly generated within the as‐designed brick‐like building wall composed of several thousand of series parallel hygro‐thermoelectric paper generators. e) The self‐powered paper house is designed to automatically generate renewable energy all‐weather from ambient hygrothermal surroundings.

The commercial filter paper employed as the flexible substrates features micro‐ and nanoscale‐sized porous fibrous configuration that is composed of abundant neighboring cellulosic fibers during a regular vacuum‐dewatering process (Figure S1, Supporting Information), endowing it perfect flexibility, accessibility, and printable adaptability compared to other glossy plastic films, brittle glass, and metal plates.^[^ ^48^, ^49^ ^]^ Among the current strategies of artificially depositing conducting materials on the surface of substrates,^[^ ^50^, ^51^ ^]^ screen‐printing is an immemorial technology but still is shown to be cutting‐edge because it has simple, cost‐effective, stable, and favorable unfailing property in many factual applications, and is especially suitable for roll‐to‐roll manufacturing. In this work, we designed an n‐, p‐type ion‐electron conductive ink and Ag electrode that are alternately printed on the surface of filter paper substrates for fabricating flexible hygro‐thermoelectric paper generator. The multi‐level pore structure inside the filter paper is very helpful to absorb and penetrate the ion‐electron ink. From the combination of different substrate materials and ion‐electron conductive ink in Figure S5b, Supporting Information, it can be stated that the combination of filter paper and ion‐electron ink shows excellent adsorption and binding properties, and the filter paper can absorb the ink well and meet the basic requirements of hygro‐thermo electric power generation devices. After absorbing moisture, the device will not cause the power generation performance to weaken due to the loss of conductive particles caused by ink overflow from the paper‐based surface. And by comparing the electrical conductivity of different types of paper‐based substrates as shown in Figure S9, Supporting Information, the electrical conductivity of three kinds of paper‐based substrates increases with temperature rise, among which the electrical conductivity of filter paper is highest, followed by copying paper and aramid paper. Since the semiconductor material used is the same, the Seebeck coefficient is also the same. According to the thermoelectric merit formula ZT = *S* ^2^ *Tσ*/*κ*, it can be calculated that the thermoelectric merit of the filter paper substrate is the largest. When the device design is consistent, the thermoelectric performance of the filter paper‐based device is the best, so the filter paper as the printable substrate is the most suitable. The ion‐electron conductive layer is embedded in the 220 µm thick fibrous filter paper substrate, but it does not damage its mechanical flexibility and strength. The as‐fabricated conductive ion‐electron ink and Ag electrodes thus produced are both partially embedded in the fibrous filter paper substrate, while the extended outer component is intrinsically integrated into an overall conductive layer by a viable screen‐printing technique (**Figure** **1**a).

Here we have proposed an HTE harvesting strategy where the moist‐diffusion, the Soret effect, and the Seebeck effect work synergistically to increase the final energy generation from ambient hygrothermal resources in the flexible paper generator. The n‐type or p‐type ion‐electron conductive ink of Bi_2_Te_3_/PEDOT:PSS matrix is modulated by highly hygroscopic glycerol as the core material, which is employed to facilitate the inorganic thermoelectric Bi_2_Te_3_ and organic thermoelectric PEDOT to generate sufficient electrons or holes as energy carries, and synchronously enable the ionization of regeneratable PSS (—SO_3_H) to release H^+^ charges as energy carries. As a result, the migration of dramatically increased H^+^ between the Ag electrodes causes the Soret effect and the moist‐diffusion effect indirectly creates an electric potential difference according to EDL theory defined as i‐HE (Figure 1b).

Our work begins with a comprehensive experimental investigation of TE, HE, and HTE to compare their electrical performance outputs under different humidity and Tg conditions utilizing a 4‐Cell figuration paper generator (Figure S2, Supporting Information). Figure 1c showed that in an extremely harsh surrounding (Tg = 0 K, RH = 10%), the paper generator only generates a weak voltage of ≈25 mV in the presence of a moist‐diffusion effect without Seebeck effect and Soret effect. This result also demonstrated that the hygroscopic glycerol‐modulated ink layer has strong moisture capture ability to regenerate PSS for ions ionization and thus diffusion even when they are deployed at extremely low humidity, which is common in dry desert or Antarctica on Earth. When increasing humidity up to about 50%, there is nearly a 2.7‐fold improvement in HE generation (≈67 mV) resulting from the increased H_2_O absorption for H^+^ charges move. However, after employing a 50 K Tg to the paper generator yet maintaining ambient humidity of 50%, the HTE generation dramatically increased up to 217 mV with about 8.6‐fold increase, which further demonstrates that the moist‐diffusion effect, Soret effect, and Seebeck effect work synergistically to boost the final voltage output. Interestingly, the HE production that tolerates the absence of the Soret effect and the Seebeck effect results in a tremendous 550 mV output with a nearly 22‐fold rise at higher humidity of 95% while preserving the integrity of their moisture‐induced ion migration.

Despite harvesting a massive amount of HE, a considerable HTE output of ≈680 mV was produced by utilizing 50 K Tg while keeping a constant high humidity of 95% (Figure 1c). These findings suggested a promising strategy for harvesting synergistically giant HTE from natural hygrothermal resources, enabling the development of the next generation of sustainable energy harvesting techniques featuring cost‐effective power devices. In this sense, it can provide a useful voltage with several or tens of volts in a hygrothermal resource with the integration of just a few cells, avoiding the current challenging integration of thousands or tens of thousands of tiny TE or HE components.

We also find that harvested HTE under low‐grade hygrothermal surroundings offers a promising future for power self‐sustained systems, particularly household power supplies. It is extremely desirable for HTE harvesting when we combine them into a regular thick wall‐brick‐like paper generator system by series–parallel connecting each paper generator unit through primarily two pathways of Style‐1 and Style‐2 and collect useful electricity either inside or outside the wall (Figure S3, Supporting Information). As is common knowledge, we generally utilize air conditioning (AC) to maintain a comfortable indoor temperature, resulting in temperature gradients between the inside and outside of the wall, while moisture within the walls continuously rises from the ambient air to maintain a certain humidity level over time. The commercial corrugated honeycomb (CH) can be used to support each n‐type and p‐type paper generator unit for the fabrication of large regular thick wall‐brick‐like generators, wherein the thick filter paper substrate is used for printing n‐type and p‐type ion‐electron ink on both sides to connect each CH (Figure S4, Supporting Information). The AC decreases the indoor temperature to a comfortable temperature of 18–26 °C when the outdoor temperature reaches a higher 28–45 °C in spring and summer, however, the indoor temperature is expected to rise to the same comfortable temperatures when the outdoor temperature falls from −40 to 0 °C in autumn and winter (Figure 1d). The Seebeck effect, Soret effect, and moist‐diffusion effect work synergistically to generate the promising HTE generation as a result of the persistent temperature gradients and humidity across the house's walls, which nearly occur throughout the entirety of the year. It was demonstrated that the application scenarios of the designed self‐power paper house comprised of several brick‐like generators can provide power for lighting or electrical devices both indoors and outdoors (Figure 1e). The hygro‐thermoelectric technique provides an exciting perspective for harvesting promising energy from natural hybridizing moisture and heat resources for self‐powered green buildings in our houses with cost‐efficiency, low‐carbon emissions, sustainability, and speedy all‐printed manufacture.

**Figure 2:**
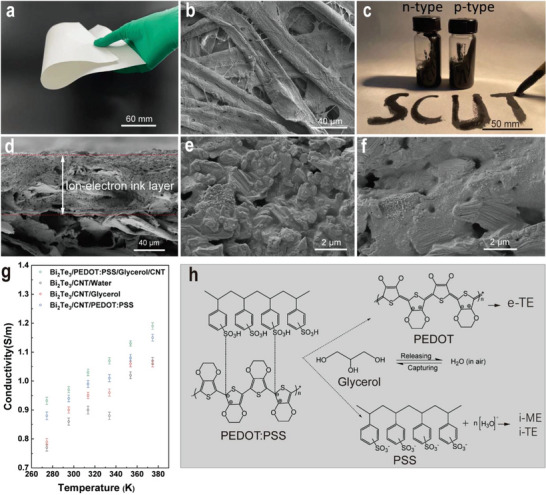
a) Digital image of the flexible filter paper substrate. b) SEM image of the paper substrate's surface with a scale bar of 40 µm; the cellulose fibers with around 25 µm width are randomly intertwined to form a porous tight structure almost without any fillings. c) Digital image of n‐type and p‐type ion‐electron conductive inks with good printing adaptability on the surface of paper substrate. d) Magnified SEM determination of the printable ion‐electron conductive layer with around 100 µm by partly embedding within the fibrous paper structure while another part is tightly adhered together. e,f) SEM images of n‐type and p‐type conductive ink, respectively, printed on the surface of filter paper substrate; it is clearly observed that Bi_2_Te_3_ particles are uniformly dispersed by glycerol and PEDOT:PSS yet still remains small porous structure. g) Comparative electrical conductivities of inks by maintaining the same Bi_2_Te_3_ use with various additive components of water, glycerol, PEDOT:PSS, and CNTs. h) Schematic illustration of the working mechanism between the glycerol, water molecular, and PEDOT:PSS for regenerating PSS and thus ionizing to produce H^+^ charges from ambient air.

Specifically, the paper substrate composed of abundant micrometer‐sized cellulose fibers has hydrophilicity, porosity, flexibility, and printing adaptability (**Figure** **2**a,b). It has been currently demonstrated that inorganic thermoelectric materials such as Bi_2_Te_3_ have a rigid and brittle nature,^[^ ^52^ ^]^ while the organic thermoelectric materials of such PEDOT:PSS are shown to be flexible and flowable.^[^ ^53^ ^]^ Although it has been shown to be an alternative strategy for yielding a comprehensive thermoelectric effect,^[^ ^54^, ^55^, ^56^, ^57^ ^]^ processing organic PEDOT:PSS and inorganic Bi_2_Te_3_ powders into a hybrid thermoelectric composite still generated unsatisfactory thermoelectric properties, which are attributed to the unclear synergistic working mechanism.

Here we first utilized high‐viscosity glycerol possessing hygroscopic and adhesive characteristics as the key material that disperses the inorganic Bi_2_Te_3_ powders, highly conductive CNTs, polyvinylpyrrolidone (PVP), and PEDOT:PSS to fabricate a bifunctional conductive ink for ion and electron transport, and more importantly enhances its printing adaptability; for example, the traditional Chinese writing brush can easily suck up the conductive ink to keep it non‐dripping, which makes it appropriate to handwriting any pattern (e.g., “SCUT”) on mostly commercial papers similar to other commercial Chinese inks (Figure 2c). In an attempt to optimize the ink's final electronic conductivity and improve the output of power generation, CNTs were supplementally utilized to enable the formation of sufficient electric conducting network within the ink layer. The experimental scanning electron microscope (SEM) results demonstrated that the conductive ink is directly printed onto the fibrous surface of flexible paper substrate, partially embedding into the fibrous structure while another part is strongly adhering together (Figures 1a and 2d). However, the majority of these works demonstrated that the electronic ink layer deposited on the surface of glossy substrates of such plastic films or flat glass it is mostly rigid, fragile and also easily delaminated due to its poor layer‐to‐layer integration with simple interface structures between the conductive layer and substrates. After being exposed to the air‐dry procedure for 24 h, the adaptability of the printed ink on rigid glossy glass and flexible PET films was far from satisfactory due to their weaker interfacial adhesives (Figure S5, Supporting Information). The embedded conductive ink structure significantly enhances strength, mechanical stability, and flexibility, and as a result guarantees highly conductive performances even when bending it at any radians. With the exception of the commercial filter paper, other flexible substrates of commercial A4 showed relatively lower porosity attributed to the fillings between the neighboring fibers; and the as‐fabricated aramid paper has poor hydrophilicity.^[^ ^58^, ^59^ ^]^

It is noteworthy that the bifunctional ion‐electron conductive ink employing n‐type and p‐type Bi_2_Te_3_ materials was defined as the n‐type and p‐type ion‐electron ink, respectively. This was accomplished while maintaining the same other significant PEDOT:PSS components, such as CNTs, PVP, and glycerol (Figure 2c). The chemical elemental structures of n‐ or p‐type Bi_2_Te_3_ material was clearly identified to its typical Sb‐doping p‐type and Se‐doping n‐type components (Figure S6, Supporting Information). Additionally, the results also showed that the size of the grinded p‐type Bi_2_Te_3_ is between 0.04 and 8.38 µm, which is far less than the size of the grinded n‐type one ranging from 0.23 to 70.88 µm (Figure S7, Supporting Information). However, they both displayed asymmetric lamella‐ or granular‐like geometrical configurations (Figure S8, Supporting Information). When the p‐type or n‐type Bi_2_Te_3_ was employed to produce the conductive ink, it was modulated by high‐viscosity liquid glycerol and PEDOT:PSS, which can efficiently disperse the Bi_2_Te_3_ powders and CNTs to form a continuous ion and electron transport network (Figure 2e,f), being different from water or solvents. The facile generation of irregular small pores throughout the resultant conductive layers is closely associated with glycerol's ability to more easily capture humidity from ambient air. The conductive ink with the optimum electronic conductivity, which increases linearly with the increase in temperature from 270 to 380 K, is shown in Figure 2g to be constituted of glycerol, Bi_2_Te_3_, PVP, PEDOT:PSS, and CNTs. However, it was found that without the use of either conductive PEDOT:PSS or the glycerol, the resultant ink displays unsatisfactory electrically conductive ability (Figure 2g). Therefore, by effectually dispersing Bi_2_Te_3_ and CNTs, the combination of the organic conductive PEDOT:PSS and glycerol promotes the facial construction of conductive structures. It is noteworthy to mention that when compared to commercial A4 paper, which is typically filled with fillings of CaCO_3_ or white clays, and aramid papers, the commercial filter paper substrate exhibited higher electronic conductivity (poor wettability for printing adaptability) (Figure S9, Supporting Information).

Although there is little evidence in the previous findings to support the essential role of glycerol in improving electronic conductivity, it is reasonable to acknowledge their tremendous potential involvements in sufficiently dispersing the conductive Bi_2_Te_3_ and CNTs materials to easily facilitate the formation of continuous electronic and ionic conductive pathway. PEDOT:PSS is another well‐known semiconductor polymer with intriguing electronic conductive properties, and more importantly, it has low thermal conductivity, is more easily soluble, and has good flexibility, making it a promising choice for use as a polymeric thermoelectric material.

Thermoelectric performances are anticipated as a result of the synergistic interactions between organic PEDOT:PSS and inorganic Bi_2_Te_3_ crystals (e.g., high Seebeck coefficient). In particular, PEDOT:PSS is currently post‐treated with organic solvents such as DMSO, DMF, NMP, or ethanol in terms of optimizing ZT value and also to help obtain a satisfactory electrical conductivity. This is attributed to the electron charges along the conjugated backbone of PEDOT chains, which makes it easier for electrons or holes to flow.^[^ ^60^, ^61^, ^62^ ^]^ Throughout the post‐treated process, the resonant structure of PEDOT chain transforms from a benzoid (B) to a quinoid (Q); and the Coulumb force interaction is produced between glycerol and PEDOT chains.^[^ ^63^ ^]^

The transport efficiency of electrons or holes is reinforced by utilizing conductive CNTs to guarantee its final electronic conductivity. When hygro‐thermoelectric paper generator works under a hygrothermal surroundings, the original conductive PEDOT:PSS conductive polymer is treated by hygroscopic glycerol instead of the previously reported DMSO, DMF, NMP, ethanol, and DMAc reagents;^[^ ^64^, ^65^, ^66^ ^]^ and the resultant PSS is regenerated to expose abundant hydrophilic —SO_3_H groups that sufficiently ionizes to release H^+^ charges in aqueous environments attributed to the accumulatively captured humidity within the porous ion‐electron ink layer by glycerol from ambient air. Here, we originally employed hygroscopic glycerol to regenerate PEDOT:PSS, which significantly increases PEDOT's electrical conductivity while also releasing the easily ionized PSS for releasing H^+^ ion charges. More importantly, the glycerol has strong hygroscopic properties that enable the porous ink layer to continuously capture humidity from ambient air, favoring the formation of continuous aqueous electrolytes along the ink layer from which the regenerated PSS with abundant —SO_3_H sufficiently ionizes to yield abundant H^+^ that diffuses across the continuous aqueous electrolyte for Soret effect and moist‐diffusion effect. Ionic transport nature is of particular interest because it offers a second feasible path to the efficient ionic moist‐diffusion effect for harvesting i‐TE from humidity under the Soret effect.^[^ ^67^ ^]^ The exceptional ink layer's ionic transport structure contributes to moist‐diffusion effect and Soret effect, but it still negatively suppresses the Seebeck effect, which produces HTE, as illustrated in Figure 2h.

**Figure 3:**
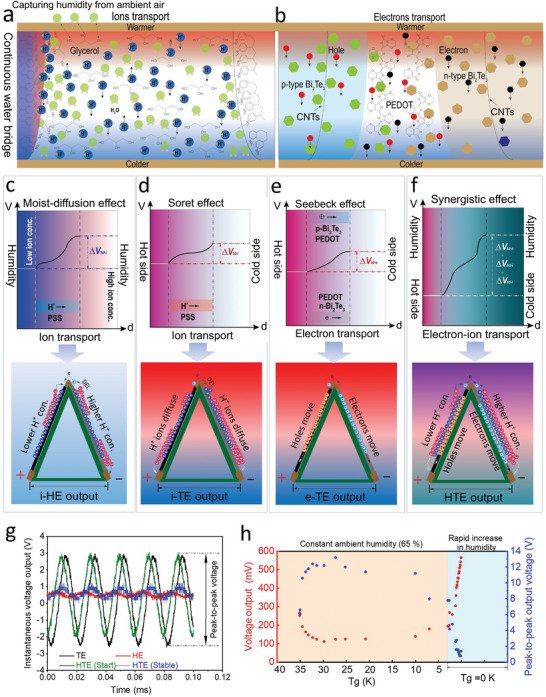
Diagrammatic mechanism of the moist‐diffusion effect, Soret effect, Seebeck effect, and their synergistic effect for different voltage outputs. a,b) Schematic diagrams of ions and electrons generation, transport, the interaction mechanisms within the i‐HE, i‐TE, and e‐TE materials under ambient humidity and temperature gradient. c) Hygroelectric potential difference (Δ*V*_hd‐i_) of ion charge carriers diagrams and the corresponding voltage (*V*) distribution of the i‐HE material by moist‐diffusion effect under ambient humidity; When the hygroelectric generation works, the difference of H^+^ ions concentration within the n‐type and p‐type ink layer induced the migration of electron from left Ag electrode‐side to right side. d) Thermoelectric potential difference (Δ*V*_td‐i_) of ions charge carriers diagrams and the corresponding voltage (*V*) distribution of the i‐TE material by Soret effect under a temperature gradient; mobile H^+^ ions of both the n‐type and p‐type ink layers will move from the top hot Ag electrode to the bottom cold corresponding Ag electrode to contribute an ionic thermo‐diffusion potential. e) Thermoelectric potential difference (Δ*V*_td‐e_) of electron charge carriers diagrams and the corresponding voltage (*V*) distribution of the e‐TE material of Bi_2_Te_3_ and PEDOT by Seebeck effect under temperature gradient. f) Synergistic hygro‐thermoelectric potential difference (Δ*V*_td‐i_, Δ*V*_td‐e_, Δ*V*_hd‐i_) of the combined electron and ion charge carriers diagrams and the corresponding final voltage (*V*) distribution of the as‐fabricated e‐TE and i‐TE materials in the two conductive ink layers by making moist‐diffusion effect, Soret effect, and Seebeck effect work synergistically under the ambient humidity–heat environments. g) Determination of instantaneous voltage outputs of the flexible paper generator by comparing the hygroelectricity (Tg = 0 K, RH = 90%), thermoelectricity (Tg = 50 K, RH = 10%), and the hygro‐thermoelectricity production (Tg = 50 K, RH = 90%). h) Experimental investigations of average voltage and instantaneous voltage outputs of the flexible paper generator were performed by gradually decreasing temperature gradients from 35 to 5 K by maintaining a constant ambient humidity of 65%, and thus rapidly increasing humidity up to much higher 90% without temperature gradient.

The bifunctional ion‐electron conductive ink structure in this work is used to generate HTE, as shown in **Figure** **3**a,b. The conductive ink layer modulated by glycerol has remarkable impacts on the ionic electric transport properties as well as the previously identified hygroscopic porous structures, as shown in Figure 2d,f. When employing the paper generator in a typical hygrothermal surrounding, the PSS is regenerated from PEDOT:PSS by hygroscopic glycerol which continuously captures humidity from ambient air, enabling the formation of continuous H_2_O bridge for aqueous electrolytes. This process further facilitates the regenerated PSS to expose abundant hydrophilic —SO_3_H groups, which thus ionizes to release a large number of mobile H^+^ charges. Because of its three hygroscopic hydroxyl groups per unit, the glycerol also directly ionizes to generate small portions of H^+^. Furthermore, the filter paper composed entirely of cellulose fibers possessing numerous hydroxyl groups can also maintain moisture to prevent its desorption.

From ambient air, humidity is ubiquitously captured to gradually accumulate within the ink layer for establishing a continuous H_2_O bridge in which the regenerated PSS sufficiently ionizes to release H^+^ (Figure 3a). However, due to the independent conductive pathway supplied by the combinable semiconductor materials PEDOT and Bi_2_Te_3_ that are strengthened by a network of CNTs, the PSS‐induced H^+^ charges process does not restrict the migration of electrons or holes for maintaining a high electric conductive ability (Figure 3b). The electrons or holes excited from PEDOT and Bi_2_Te_3_ only travel from the hot‐side (Warmer) to the cold‐side (Colder) under a certain Tg to produce thermoelectric power.

In order to expedite the synergistic humidity and heat conversion up to a significant power output, both ions and electrons employed as energy carriers can be safely transported within the bifunctional ion‐electron conductive layer structure with high performances. In this regard, the migrations of ions and electrons inside the conductive ink layer serve as important pillars in generating HTE by integrating the synergistic interactions of moist‐diffusion, Soret effect, and Seebeck effect. The diagrammatic mechanisms of the i‐HE (moist‐diffusion effect), i‐TE (Soret effect), e‐TE (Seebeck effect), and their synergistic HTE under various conditions are illustrated in Figures 3c–f, respectively. The continuous water‐bridges that are accumulated in the n‐type and p‐type ink layer structures carried the H^+^ charges released from the abundant active —SO_3_H in the regenerated PSS matrix to move.

According to Figure 2e,f, the size of n‐type Bi_2_Te_3_ particles is much larger than p‐type Bi_2_Te_3_ particles and, also much more pores and surface areas per unit volume are observed within the n‐type ink layer (Figures S7 and S8, Supporting Information). This results in an increased exposure of hygroscopic glycerol and PSS to ambient air, which accelerates humidity absorption and thus releases increased H^+^ charges for enlarging its effective diffuse pathways. Additionally, the above deduction was experimentally demonstrated by that the n‐type ink layer seems easier drying and hygroscopic compared to the p‐type one even when the same ambient humidity is employed. The formation of a dynamic stable EDL structure and the migration of dramatically increased electrons from the Ag electrode‐side (nearby the n‐type ink) to another side is induced by the much higher H^+^ ions concentration that, when only the moist‐diffusion for hygroelectric generation is operating, will dissociate from PSS within the n‐type ink layer and diffuse to accumulate adjacent to the interfaces of the top Ag electrode (nearby the p‐type ink). In terms of generating a dynamic, stable EDL structure, the mobile H^+^ ions within the p‐type ink layer will diffuse onto the interface of the Ag electrode‐side (adjacent to the p‐type ink). In order to establish a diffusion current and potential difference between the left and right Ag electrodes for generating electricity, mobile H^+^ ions will diffuse from p‐type ink layer to n‐type ink layer (Figure 3c). Figure 3d illustrates that when only the Soret effect is working, mobile H^+^ charges from the n‐type and p‐type ink layers will move from the top hot‐side to the bottom cold‐side of the corresponding Ag electrodes, respectively. This enables an ionic thermo‐diffusion potential because of the presence of a difference in the concentration of H^+^ ions. The Seebeck effect works by transferring electron or hole charges dissociated from Bi_2_Te_3_ and PEDOT migration from top hot‐side Ag electrode to the bottom two cold‐sides of the corresponding Ag electrode and thus generates a thermoelectric potential between them. This is in way of comparison to the moist‐diffusion effect and Soret effect induced by diffusive ions (Figure 3e). When the paper generator operates in a hygrothermal environment, an elegant approach is used to make the i‐HE (Δ*V*_hd‐i_), i‐TE (Δ*V*_td‐i_), and e‐TE (Δ*V*_td‐e_) production collaborate to boost the final HTE outputs owing to synergistic ion‐electron transport (Figure 3f).

By comparing the onefold TE generation (Tg = 50 K, RH = 10%), the HE generation (Tg = 0 K, RH = 90%), and the HTE generation (Tg = 50 K, RH = 90%, at starting stage and stable stage, respectively) with a Tektronix Oscilloscope measurement, the detailed experiments were performed to monitor the variations of instantaneous and peak‐to‐peak voltage output signs, as shown in Figure 3g. At 50 K Tg and 10% RH, the instantaneous voltage of onefold TE generation exhibited typical fluctuating sine waveforms with the peak‐to‐peak voltages ranging from −3.0 to 3.0 V. However, the HE voltage displayed much smaller sine waveform fluctuations, with much smaller peak‐to‐peak voltage ranging from 0 to 1.0 V, when ambient humidity dramatically increased to 90% without employing any Tg. This can be explained by the fact that HE is generated indirectly by employing ions as energy carriers through a DEL pathway, whereas TE is generated directly by employing electrons or holes as energy carriers. However, the HTE voltage showed the similar trend to the TE voltage in the initial stage, and then to the HE voltage in the stable stage with a larger peak‐to‐peak voltage (Figure 3f). Further experimental investigations were carried out by determining the average voltage and instantaneous voltage outputs of the paper generators by gradually decreasing temperature gradients from 35 to 5 K by maintaining a constant ambient humidity of 65%, and thus rapidly increasing humidity to much higher 90% without temperature gradient (Figure 3h), the voltage output will also increase rapidly than the normal humidity. Because the density of water molecule is higher in high humidity, the paper generator can absorb a large amount of moisture, so the ion‐electron conductive ink can dissociate freely mobile H^+^, the device can form more continuous water bridge structures to promote the migration of ions (Figure 3a), ions move with less resistance, under the moist‐diffusion effect (Figure 3c) and Soret effect (Figure 3d), the rate of ion migration will also be faster, so the rate of voltage rise under high humidity is higher than under normal humidity and lower humidity. The results demonstrated that the HTE generation displays a different parabolic variation tendency compared to the typical TE or HE production. With the decrease of Tg yet maintaining a constant 65% humidity, the HTE output first descends and thus arises slowly while the peak‐to‐peak output shows an opposite pattern. However, the HE output shows a sharp increase from 194 to 565 mV without any temperature gradients.

**Figure 4:**
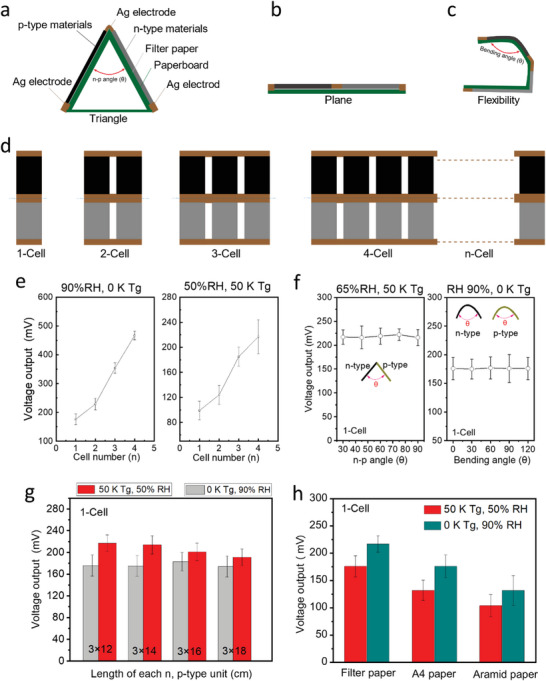
a–c) Schematic diagrams of the paper generator with different flexible or plane configurations. d) Hybrid mode of different series parallel devices with 1, 2, 3, 4, and *n* cells. e) Impacts of the number of cells within a flexible paper generator on the HE generation under 90% RH and 0 K Tg, and also the HTE generation under 50% RH and 50 K Tg. f) Influences of the bending angles of Ag electrode as well as the n‐type and p‐type ink layers on the final voltage generation from 1‐Cell device. g) The influences of cell's length on the hygroelectric and hygro‐thermoelectric generation; and h) the comparative voltage outputs of filter paper, A4 paper, and aramid paper substrates under Tg (0 and 50 K) and humidity (50% and 90%).

We alternately printed n‐type and p‐type ion‐electron conductive ink and Ag electrodes on a flexible filter paper substrate to design a triangle‐shaped Ag electrode configuration that can be sandwiched between hot‐side and cold‐side Ag electrodes (**Figure** **4**a–d). As demonstrated in the previous discussions, it is crucial that bifunctional ion‐electron conductive ink or an Ag electrode be directly printed onto the fibrous paper substrates to form an integrated functional structure, rather than on a glossy glass or plastic substrate, thereby enabling a highly stable conductive ink layer and guaranteeing its ionic and electronic conductivities while still maintaining perfect flexibility and foldability (Figure 4a–c). The flexible paper generator is configured with a variety of series–parallel Ag electrodes connections to construct a cell unit (1‐Cell), two cell units (2‐Cell), three cell units (3‐Cell), four cell units (4‐Cell), or many cell units‐figurate paper generators (*n*‐Cell) (Figure 4d). The distance between each cell unit is 2.0 cm, and the design of the triangle paper generator is as simple as folding the paper in half along the central Ag electrode part (Figure 4a). In addition, the increase in cell count in the paper generator undoubtedly enhances the HTE generation.

Figure 4e showed that the additional experimental investigations were conducted utilizing various cell counts under two different settings. Owing to the gradually increased ions transport regions, the i‐HE output increased linearly from 176 to 467 mV from one cell (1‐Cell) to four cells (4‐Cell)‐figurate paper generators at 90% relative humidity when the temperature gradient was kept constant. In particular, the HTE output of paper generators ranging from 1‐Cell to 4‐Cell increased dramatically from 99 to 217 mV when 65% relative humidity and 50 K Tg were employed (Figure 4e).

To investigate the impacts of flexibility on electrical outputs, a few 1‐Cell‐figurated paper generators were bended with different various angles of the top middle Ag electrode between n‐type and p‐type components, as well as the different angles of either n‐type or p‐type part under the same components and conditions (Figure 4f). Moreover, the results revealed that the HTE outputs (about 218 mV) were mostly unaffected even when adjusting the n–p angles by 30°, 45°, 60°, 75°, and 90° under appropriate conditions of 65% relative humidity and 50 K Tg, and the final output stability was around 175 mV regardless of whether the bending angle of the n‐type or p‐type ink layer was 0°, 30°, 60°, 90°, or 120°, indicating the perfect printable stability of commercial Ag electrode ink on filter paper substrates besides our as‐fabricated ion‐electron conductive ink. The promise of flexibility and stability of the printable inks was attributed to the unique integrated imbed‐structure conductive ink layer supported by the fibrous paper substrates composed of numerous neighboring cellulose fibers and porous structures.

Due to the different distances at which ion, electron, or hole charges travel between the Ag electrodes, the length of each n‐type and p‐type cells has an impact on HTE production with a 1‐Cell. Figure 4g demonstrates that increasing the length of each cell from 12.0 to 18.0 cm has even less effect on HE generation at 90% RH and 50 K Tg, but has a slight impact on TE generation at 50% RH and 50 K Tg. Moreover, commercial filter paper offers superior electrical conductivity and flexibility compared to commercial A4 and aramid paper, as well as superior hydrophilicity and fewer fills, despite their comparable printability. Figure 4h demonstrates that the filter paper generated higher HE (90% RH) and HTE (65% RH, 50 K Tg) than the hydrophobic aramid paper substrate, where voltage outputs were unsatisfactory.

The above mentioned had demonstrated that the moist‐diffusion effect and the Soret effect had an increased impact on the final HTE outputs compared to the Seebeck effect. This can be explained by the fact that dramatical ions migration accelerates the formation of giant potential differences, whereas electronic migration requires either the challenging integration of sufficient conductive pathways or the Seebeck effect. However, despite considerable efforts, little study has been undertaken into their ability to work synergistically to harvest final hygro‐thermoelectric power. Importantly, the simultaneous H^+^ diffusion for i‐TE and i‐HE within the ion‐electron conductive ink layer was observed to positively boost e‐TE generation during HTE generation.

As identified, the generation pattern of humidity in ambient environments determines the efficiency and pathway of H_2_O capture by glycerol within the conductive ink layer, which has a major impact on H^+^ ionizations and diffusions. Our study assessed the following two different patterns for humidity generation: 1) installing the testing chamber with a sealed plastic chamber (0.01 m^3^) to which the appropriate amounts of water are added to ensure the natural evaporation of H_2_O to gradually adjust the balance of relative humidity in the chamber (Figure S10a, Supporting Information), as defined by Evaporation‐1; 2) utilizing the artificial nebulization of water by a Mistorizer to rapidly adjust the relative humidity in the chamber (Figure S10b, Supporting Information), as defined by Evaporation‐2. Additional temperature gradient is combined to the paper generator for various measurements when adjusting humidity in the two moisture production patterns discussed above.

As depicted in Figure S11, Supporting Information, the HE generation in the Evaporation‐1 pattern significantly increased from 310 to 450 mV as the relative humidity rose from 73% to 87%. However, the peak‐to‐peak output voltage displayed a decreasing trend from 1.70 to 0.75 V. By initiating the Evaporation‐2, the output voltage increased abruptly to a higher 533 mV at the increased humidity of 94%, and then gradually increased to 548 mV even when humidity reaches 100%, at which point the peak‐to‐peak voltage output decreases to a much lower level of ≈0.3 V. Overall, the above results demonstrated that humidity from ambient air has the perfect ability to modulate the ionizations of PSS to generate H^+^ charges as the energy carrier for HE production. However, different characteristics of electric outputs were observed in Evaporation‐1 and Evaporation‐2 due to their distinct water molecular supplies. As we can see, the Seebeck effect is almost exclusively attributed to the migration of electrons or holes in e‐TE generation, with even less influence from humidity.

**Figure 5:**
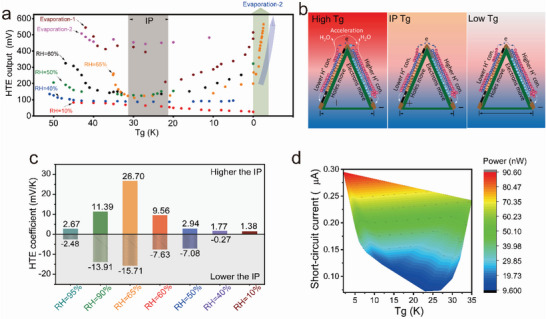
Electric generation of the hygro‐thermoelectric paper generator. a) Experimental determinations of e‐TE outputs under 10% relative humidity, and HTE outputs under humidity (40–95%) and Tg (0–50 K). b) Schematic illustrations of ions and electrons transport mechanism for HTE generation under different Tg conditions. c) Comparative studies of HTE coefficients under different humidity ranging from 40% to 95%. d) The Contour layouts of describing short‐circuit current and power of HTE generation under 63% humidity by changing Tg from 0 to 35 K.

The output of e‐TE generation decreased linearly from 83 to 25 mV as Tg decreased from 45 to 0 K in an extremely low humidity of 10% (**Figure** **5**a), indicating a conventional e‐TE generation pattern. Nonetheless, it shows a Seebeck coefficient of ≈1.38 mV K^−1^, which exceeds the majority of previously reported works.^[^ ^68^, ^69^ ^]^ If Tg is performed by maintaining a higher relative humidity, both ion and electron charges travel concurrently and synergistically within the conductive layer to generate HTE. Additional experiments were conducted to determine their synergistic working mechanisms utilizing 10–95% relative humidity, and temperatures range from 10 to 50 °C (Figure 5a). However, the open‐faced parabolic trend was clearly observed in HTE generation under changing Tg (0–50 K) in response to the various humidity (40–95%). At the beginning, under the condition of higher Tg, carriers migrate rapidly, the Seebeck effect is significantly higher than the moist‐diffusion effect and the Soret effect, the Seebeck effect plays a major role in the output voltage at this time, so at the beginning, the voltage decreases with the drop of Tg (Figure 5a), indicating a conventional e‐TE generation pattern. And when the temperature decreases to the IP region, the Seebeck effect, the moist‐diffusion effect, and the Soret effect reach equilibrium, at which time there is no large change of the voltage output. As the temperature is further reduced, the moist‐diffusion effect and the Soret effect are significantly higher than the carrier migration, the moisture induction plays a key role in this stage. Thus, as the Tg decreases, the evaporation rate of moisture decreases and the paper generator starts to gradually capture moisture spontaneously, while the rate of the generator capturing moisture accelerates as the temperature difference decreases, thereby releasing more free‐moving ions, lead to the voltage increases in the post stage.

Specifically, there is always a regular “Inflection Point (IP)” region at Tg between 22 and 32 K, during which the final HTE generation displays only a slight fluctuation regardless of the utilization of different relative humidity. When Tg is higher than IP, HTE generation decreases gradually with decreasing Tg, whereas the opposite is happening when Tg is less than IP. For example, it was revealed that as relative humidity increased from 40% to 65%, the resultant parabolic curve became steeper, resulting in a higher voltage output at the same Tg. When a higher Tg is employed, the accelerated water evaporation within the upper ink region close to the top hot Ag electrode not only increases the released H^+^ magnitudes and the migration efficiency, but also helps alleviate the negative effects of ions migration within the p‐type ink layer on the final electric potential generation of the bottom cold positive electrode by the Soret effect (Figure 5b). Increased humidity reinforces the synergistic HTE generation arising from the interaction of the Soret effect, moist‐diffusion effect, and Seebeck effect.

Less change in HTE generation was observed in the “IP” region, which was attributed to the excessive ions charge migration within the p‐type ink layer by the Soret effect from the top hot Ag electrode to the bottom cold one, almost counteracting the combined performance of the positive Seebeck effect and the moist‐diffusion effect, even when the relative humidity changed from 40% to 65% (Figure 5a,b). However, it was observed that HTE production significantly increases as Tg falls below the IP region of 0–25 K. This result can be explained by the fact that water evaporation was drastically reduced to almost nonexistent, and the Seebeck effect and Soret effect become slightly impactful; therefore, the moist‐diffusion effect plays a crucial role in generating HTE (Figure 5b). When decreasing Tg closer to 0 K, the final HE output rises rapidly from 194 to 565 mV (Figure 5a), when the ambient humidity of 65% rapidly rises to a higher 95% by Evaporation‐2, demonstrating the superiority of the moist‐diffusive effect in achieving massive final voltage outputs. Therefore, we can conclude that humidity has a greater impact on the final power output. Extremely low or no moisture, there is no free mobile ion charges to dissociate, so the moist‐diffusion effect, the Soret effect cannot provide power, resulting in much lower power as shown in Figure 5a. Tg has less impact on the power outputs when employing extremely lower relative humidity of 10%. i‐TE energy generates a 100 times higher thermal voltage than e‐TEs,^[^ ^19^, ^67^ ^]^ and the largest contributions of HTE performance are mainly the moist‐diffusion effect and the Soret effect, while the Seebeck effect is based on temperature difference conditions can be less effective. The number of free mobile ion charges in the ion thermoelectric material determines the ion thermal voltage, and the change of humidity affects the number and mobility of free moving ions H^+^ within the ion‐electron conductive ink, so the humidity plays a major role in on power generation effect.^[^ ^70^ ^]^ In addition, for the moist‐diffusion effect, humidity also has a significant effect on the internal migration of free ions. Thus, both effects that contribute significantly to HTE power are based on the migration of free mobile ions (Figure 3a), while the external humidity determines the number and mobility of free mobile ions, and thus the humidity has more influence on the final power output.

It is worth noting that when using a higher relative humidity in the Evaporation‐1 and Evaporation‐2 patterns in the testing chamber, the HTE showed a similar, albeit shallower, tendency of the parabolic curves as a result of varying Tg, and still produced a significantly higher HTE output than those from a lower relative humidity. In particular, in the extremely high humidity conditions of 95–100% in Evaporation‐2, the HTE exhibited a higher voltage output with less fluctuation than in Evaporation‐1. This result demonstrated that the combined ionic Soret effect and moist‐diffusion effect will play a vital role in the performances of HTE in surroundings with considerably higher humidity.

Similar to conventional e‐TE, i‐TE, or i‐HE materials, the synergistic voltage generation resulting from ions and electrons as energy carriers is defined as HTE power; however, it is necessary to clarify our basic terminologies because no report has been published regarding its definition. HTE coefficient is defined as the slope of this linear fitting equation produced from the final HTE voltage outputs to Tg. When Tg is higher than the IP region, the HTE generation decreases as Tg falls in a positive manner; whereas, when Tg is lower than the IP region, the HTE generation increases as Tg decreases in a negative manner. As depicted in Figure 5c, the positive HTE coefficient of the paper generator reaches a maximum of +26.7 mV K^−1^, while the negative HTE coefficient reaches a maximum of −15.71 mV K^−1^ at an average humidity of 65%. This result indicated that our as‐fabricated paper generator can be used to harvest energy with the optimum performance in typical domestic settings. Extremely high or low humidity contributes negatively to the HTE coefficient; for example, 95% humidity only results in a positive +2.67 and negative −2.48 mV K^−1^ HTE coefficient, while it can provide the optimum HTE output level of roughly 680 mV (Figure 5c). Decreasing humidity from 65% to 40% decreases the HTE coefficient considerably in both positive and negative styles. We see that the Seebeck coefficient of the paper generator reaches up to 1.38 mV K^−1^, which eliminates the effects of the extremely low relative humidity of ≈10%.

In addition, we investigated the variations in short‐circuit current and power outputs of the paper generator at 63% relative humidity and Tg values ranging from 0 to 35 K, as shown by a Contour diagram. Similar to the HTE voltage outputs, the short‐circuit current outputs appeared to display a parabolic relationship with Tg; however, the power outputs were enhanced when a lower Tg was employed (Figure 5d). At 2.0 K Tg, it shows a higher short‐circuit current of 0.3 µA and a power of 90.6 nW. When removing the temperature gradient and subsequently employing an Evaporation‐2, additional experimental results revealed that the short‐circuit current sharply increased to 0.704 µA with the corresponding power of 397.8 nW, which exhibited higher outputs than those by employing a high Tg of 30–35 K, indicating that the ionic moist‐diffusion effect has a noticeable effect on the outputs of short‐circuit current and power. The improved synergistic moisture‐heat transfer efficiency as a result of the broad moist‐diffusion effect, the Soret effect, and the Seebeck effect, as well as the improved ions and electrons management, demonstrated by these results, illustrates the material's enormous potential for HTE generation.

## 3 Conclusion

We demonstrated a novel HTE harvesting strategy that utilizes the natural low‐grade hygrothermal resources to synergistically make the Seebeck effect, Soret effect, and moist‐diffusion effect work together, thereby generating giant final power outputs for future self‐powered houses and apartments. The flexible HTE paper generator was directly processed by fully printing ion‐electron conductive ink and Ag electrodes onto commercial paper substrates to form a unique ionic electronic transport structure using PEDOT:PSS/Bi_2_Te_3_ as the basic matrix which is modulated by hygroscopic glycerol to regenerate ion providers (PSS) for moist‐diffusion effect and Soret effect, as well as electron or hole providers (Bi_2_Te_3_, PEDOT). The experimental results demonstrated that the i‐HE, i‐TE, and e‐TE can work synergistically to boost HTE generation under regular hygrothermal conditions. The as‐fabricated flexible paper power device offers opportunities for the design of self‐powered paper houses with low preparation costs, a simple process, low carbon emissions, and sustainability. Furthermore, it can overcome the existing bottleneck problem of being unable to naturally utilize waste heat and moisture in regular factual environments to enable them to work together. However, there are also some difficulties and challenges of this project. First, the actual movement and migration of ions and carriers in ion‐electron conductive ink under various impacts is complex and lack the appropriate technical means of detection; we only follow the experimental results of possible ion and electron carriers’ movement to elaborate. Besides, the current output of our ion‐electron conductive ink is still relatively low, and the overall output power is also not ideal, which needs to be further strengthened and improved for the actual production and life applications. Finally, the prospects for expanding the application of ion‐electron conductive ink on other inorganic building materials are not yet known. Nevertheless, this work is still a groundbreaking investigation into the harvesting of massive HTE that can meet the urgent power demands for green, low‐carbon, and personalized development in the process of people's lives and industrial productions, allowing self‐powered devices to operate in a new way.

## 4 Experimental Section

### Chemicals and Materials

All the chemicals used in this study were analytical grade and directly used without any further purifications. The glycerol (99.0%, AR, C_3_H_8_O_3_) was purchased from Shanghai Runjie Chemical Reagent Co. Ltd. n‐type (Se‐doped) and p‐type (Sb‐doped) bismuth telluride crystal (Bi_2_Te_3_) powders (99.0%, AR) were kindly obtained from Hangzhou Kaida Co. Ltd. PEDOT:PSS (99.0%, AR) and PVP K30 with 99.0% purity and analytical grade were purchased from Shanghai Boao Biotechnology Co. Ltd. Multi‐walled CNTs (95.0%, AR) with the diameter less than 8 nm and length of 0.5–2.0 µm was purchased from Nanjing Xianfeng Nanomaterials Technology Co. Ltd. The quick drying‐type conductive electrode of silver paint (66.0%, AR) was kindly supplied by Guangzhou KaiXiang Electronic Products Co. Ltd. The 0.2 mm thickness commercial filter paper with basic weight of 120 g m^−2^ was purchased from Suzhou Baweide Environmental Protection Technology Co. Ltd.

### Preparation of Ion‐Electron Ink

Either n‐type or p‐type bismuth telluride crystals’ (Bi_2_Te_3_) powders was, respectively, ground into smaller size, followed by air‐drying process at 80 °C for 24 h. The resultant 400.0 g n‐type or p‐type Bi_2_Te_3_ powders was, respectively, mixed with 400.0 g glycerol and 100.0 g PVP for at least 30 min, and thus added by dropwise 0.1 g PEDOT:PSS solution for another stirring process at a high‐speed of 1500 rpm, followed by additional ultrasonic treatment for 15 min using ultrasonic cleaner (JP‐020S, Shenzhen JieMeng). Therefore, the n‐type and p‐type ion‐electron ink were, respectively, obtained after a deaeration process.

### Fabrication of Substrate

The commercial filter paper (*d* = 0.2 mm, weight = 81.29 g m^−2^) was cut into rectangular paper substrate with 3.0 cm width and 12.0 cm length piece as the screen‐printing substrate; and another commercial white cardboard with 0.5 mm thickness was employed to support the filter paper printed using ion‐electron ink process. The commercial double sticky tape was used to glue tightly the filter paper and paper white card together. The substrate was divided into five areas of 1, 4, 2, 4, and 1 cm length in the length direction for printing ion‐electron ink and Ag electrode.

### Coating of conductive ink

Then 1.2 g of p‐type and n‐type conductive ink was, respectively, screen‐printed on the two 4 cm area, conductive silver paste on the 1 and 2 cm area, high precision high conductivity copper wire with conductive adhesive was pasted on both ends of the conductive substrate and dried at room temperature for 180 min to make the paper generator. Therefore, n‐type and p‐type ink was connected by Ag electrode to form a paper generator with a series–parallel electric circuit structure. The printable weight of conductive ink per square centimeter on paper substrate was 0.1 g and the weight of Ag electrode was 0.05 g. The highly conductive copper wire was connected to the Ag electrode on both ends using a conductive Ag adhesive to output electric sign for determinations. The resultant paper generator was pretreated under various humidity and Tg conditions for investigating i‐ME, i‐TE, and e‐TE, and hygro‐thermoelectric production, respectively.

### Measurements

Using a sealed plastic container of 50.0 cm (length) × 30.0 cm (width) × 30.0 cm (height), the punched hole on each side of the container was utilized to enable moisture or/and air inflow and also outflow to control humidity; and another sealed hole on upper side of the container was used for the real‐time determinations of electric outputs and humidity, respectively, performed by Tektronix oscilloscope (TBS1202B, Tektronix) and hygrometer (DT‐8896, Huashengchang). One pathway to obtain relative humidity within the sealed container was modulated by natural evaporation of the added liquid water below the container; and another pathway of compulsive water nebulization was employed to artificially enhance rapidly the humidity up to a much higher lever through an ultrasonic atomizer (WH‐2000, Guangdong Yuehua). A triangular configuration possessing n‐type and p‐type ink printed on each side was utilized for determining the hygro‐thermoelectric outputs. Herein, the supplied warmer was exerted on upper side of Ag electrode while the colder was exerted on bottom side of Ag electrodes to establish a temperature gradient field. The warmer or colder resource was supplied using the sealed glass cylinders with 23.0 cm (length) × 10.0 cm (width) × 12.0 cm (height) containing hot or cold water; the temperature collector (YP‐5008G, Shenzhen Yongpeng) was used to real‐time monitor temperature changes on both hot‐side and cold‐side.

### Characterization

The particle size distribution of n‐type and p‐type Bi_2_Te_3_ powders was analyzed by Master sizer 3000 laser particle size meter (JL‐6000, Chengdu Jingxin, China). Using Merlin field emission SEM (ZERSS, Germany) at an acceleration voltage of 5.0 kV, the surface morphology of the semiconductor Bi_2_Te_3_ powders and the ion‐electron ink layer was observed. The electric conductivity of the paper generator was tested with a four‐probe square resistance tester (KDY‐1, Guangzhou Kunde) by considering the fractional contribution to conductivity of the hybrid ion‐electron ink. Tektronix oscilloscope (TBS, Tektronix) was used to test the short‐circuit voltage output of hygroelectricity, thermoelectricity, and also HTE, respectively. The X‐ray photoelectron spectroscopy was measured by Kratos Axis Ultra DLD electron spectrometer to determine the distributions of main elements of Bi_2_Te_3_ materials. Real‐time humidity was recorded by utilizing a humidity meter CEM DT8896.

## Conflict of Interest

The authors declare no conflict of interest.

## Author Contributions

H.S. and K.X. contributed equally to this work and should be regarded as co‐first authors. H.S. and K.X.: Methodology, software, and data curation. H.S.: Writing—original draft preparation. K.X., Y.D., and P.W.: Visualization and investigation. D.L.: Supervision and conceptualization. Z.Q., Y.C., Y.Lu., and C.L.: Software and validation. Y.L. and J.C.: Writing–reviewing and editing.

## Supporting information

Supporting Information

## Data Availability

The data that support the findings of this study are available on request from the corresponding author. The data are not publicly available due to privacy or ethical restrictions.
