# Supplementary material for: All‐Printed Flexible Hygro‐Thermoelectric Paper Generator
Source: Adv Sci (Weinh). 2023 Jan 22;10(9):2206483. doi: 10.1002/advs.202206483 (PMC10037691; doi:10.1002/advs.202206483)
Supplement: Supplementary file 1 — Supporting Information [file ADVS-10-2206483-s001.pdf]

Supplementary information for

All-printed Flexible Hygro-thermoelectric Paper Generator

Haoyu Shen¶, Ke Xu¶, Yulong Duan, Peilin Wu, Zhiyun Qian, Yonghao Chen, Yao Luo, Chaocheng Liu, Yang Li, Jiedong Cui, Detao Liu*

School of Light Industry and Engineering, State Key Laboratory of Pulp and Paper Engineering, South China University of Technology, Wushan Rd., 381#, Tianhe District, Guangzhou, Guangdong 510640, China

The email address of corresponding author: dtliu@scut.edu.cn

This Supplementary information file includes:

Figures S1 to S11

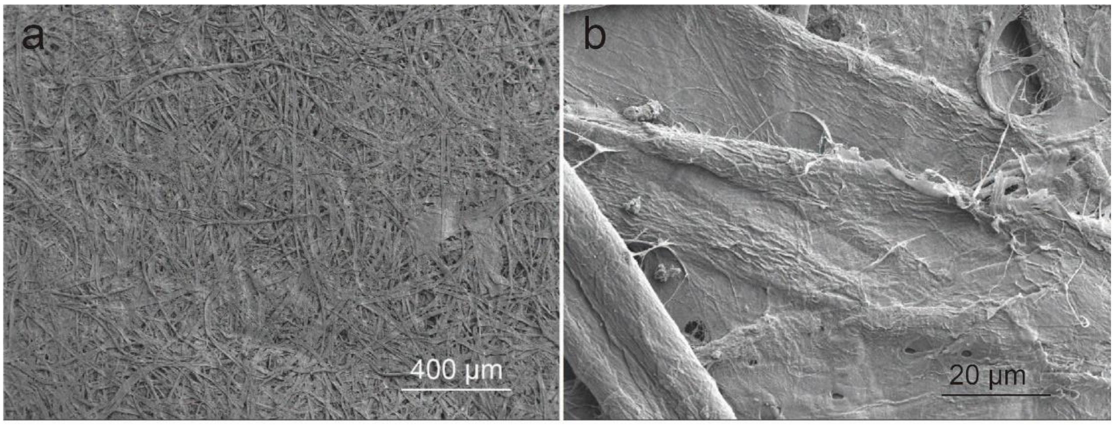

Figure S1 SEM image of the commercial filter paper by 400 μm scale bar **(a)** and 20 μm scale bar **(b).**

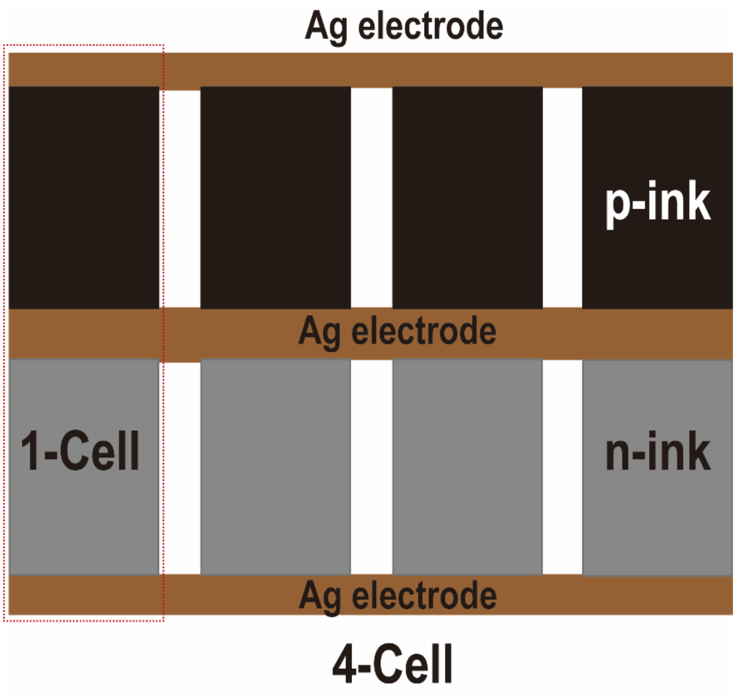

Figure S3. Schematic profile of filter paper-based generator with 4-Cell.

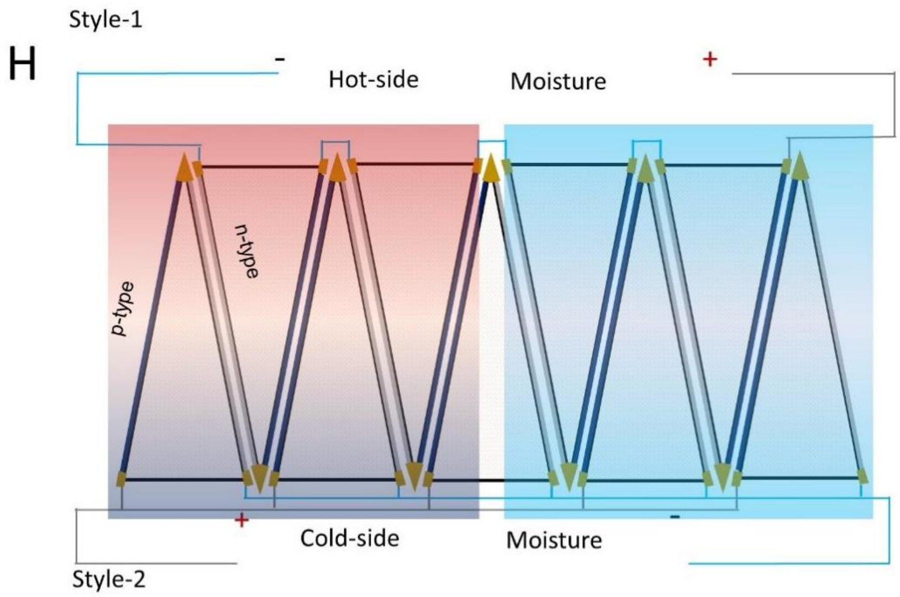

Figure S3. Schematic program of series-parallel connecting each paper generator unit through primarily two pathways of Style-1 and Style-2.

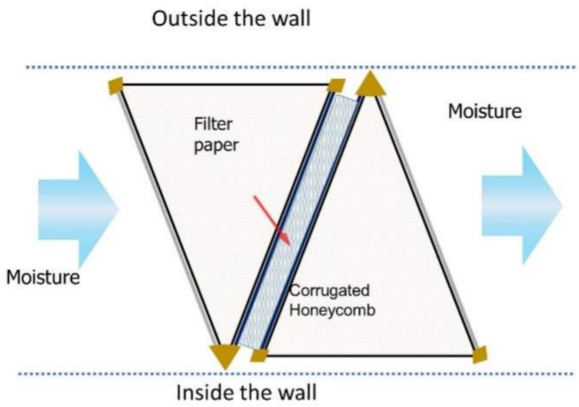

Figure S4. Schematic program of series-parallel connecting each paper generator unit through primarily two pathways of Style-1 and Style-2.

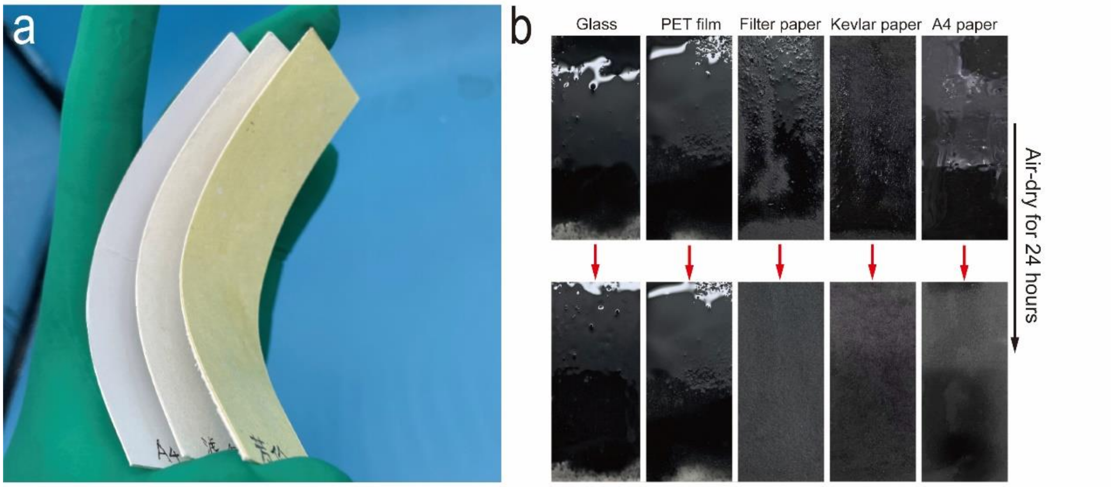

Figure S5. Comparative printing adaptability of the bifunctional ion-electron conductive ink on different Glass, PET film, Commercial A4, filter paper and Artificial aramid paper substrates through a 24 hours air-dry process.

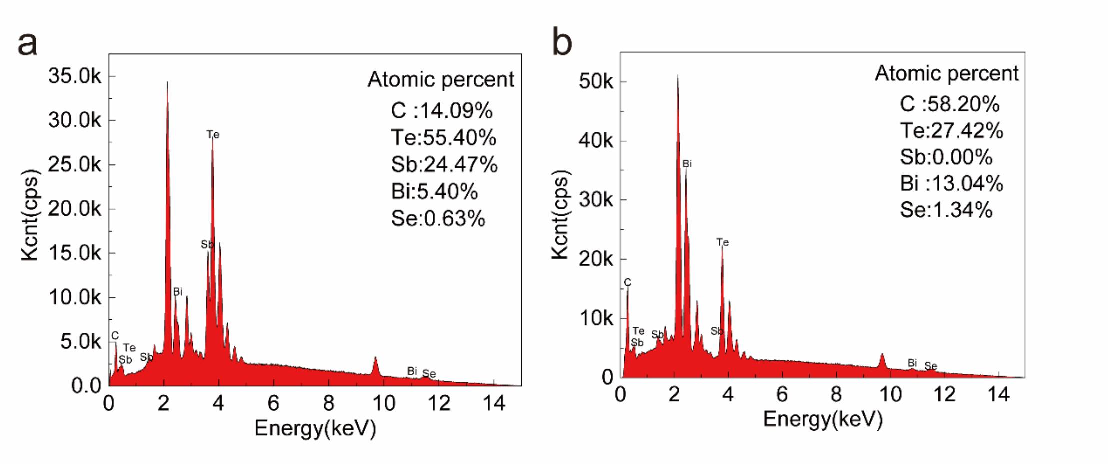

Figure S6. Chemical elemental identifications of p-type (a) and n-type Bi_2Te_3 (b) materials were carried out to further demonstrate its typical components with semiconductor structures (p-type and n-type).

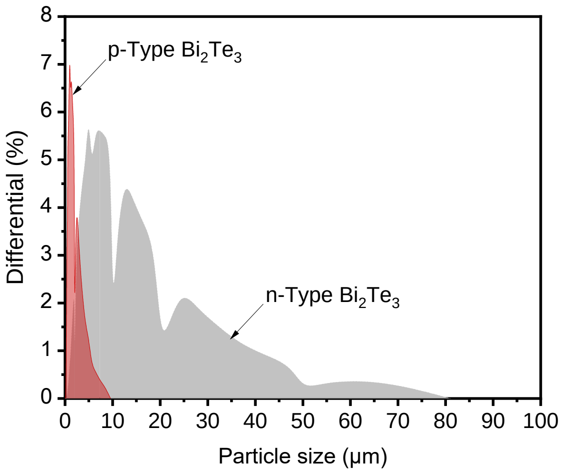

Figure S7. Size determinations of Bi_2Te_3 powders with p-type and n-type by Master sizer 3000 laser particle size meter.

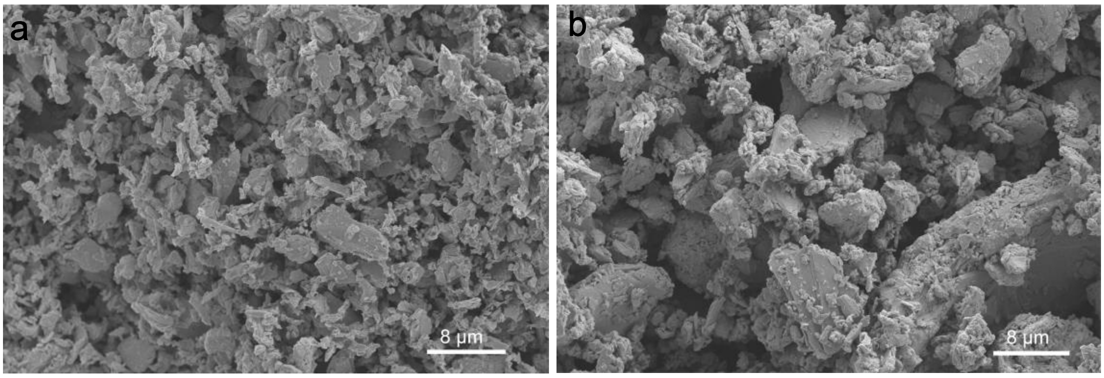

Figure S8. SEM images of Bi_2Te_3 powders with p-type (a) and n-type (b) by Merlin Field Emission Scanning Electron Microscope.

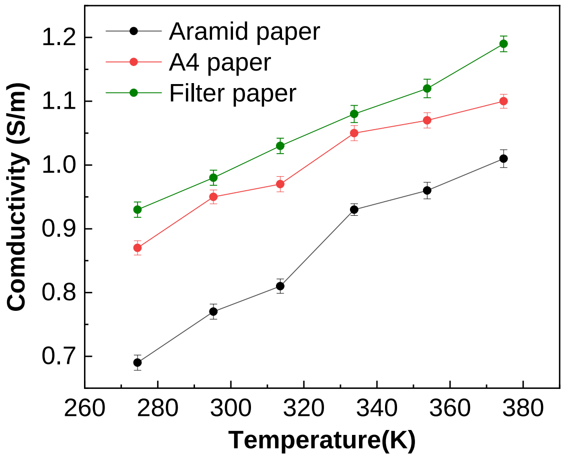

Figure S9. Comparative studies on electronic conductivity of screen-printing ion-electron ink onto the different substrates of Aramid paper and the commercial A4 paper and Filter paper.

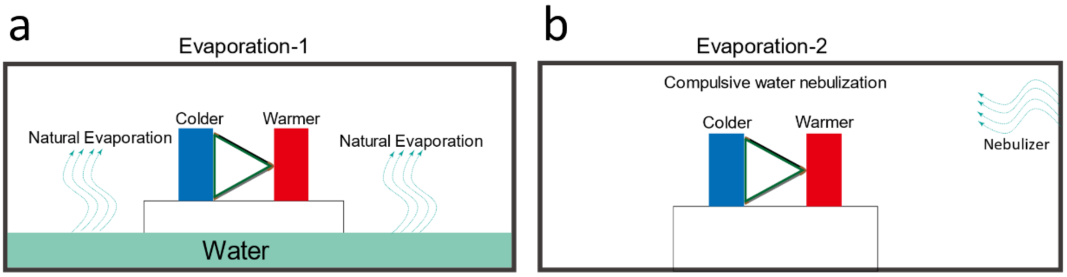

Figure S10. (a) Humidity raising conditions by natural water evaporation defined as “Evaporation-1”. (b) Compulsive water nebulization defined as “Evaporation-2”.

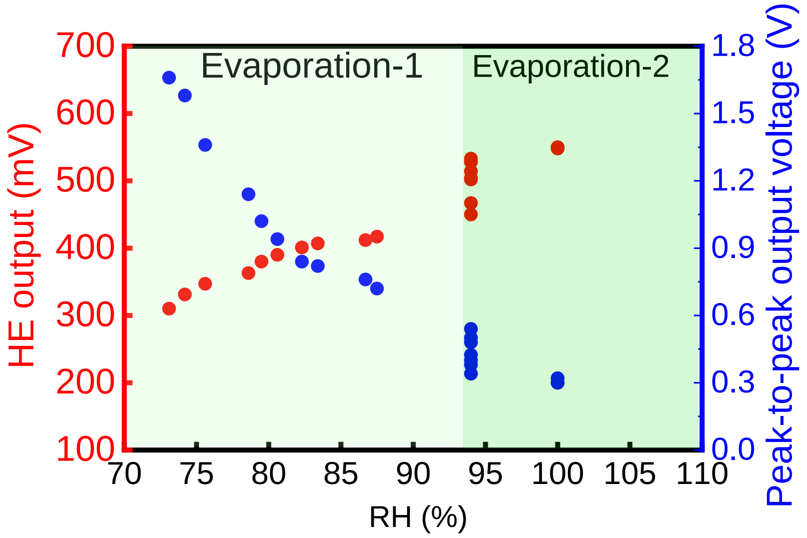

Figure S11. Comparative investigations on HE outputs in the initial Evaporation-1 stage and subsequent Evaporation-2 stage without temperature gradient.

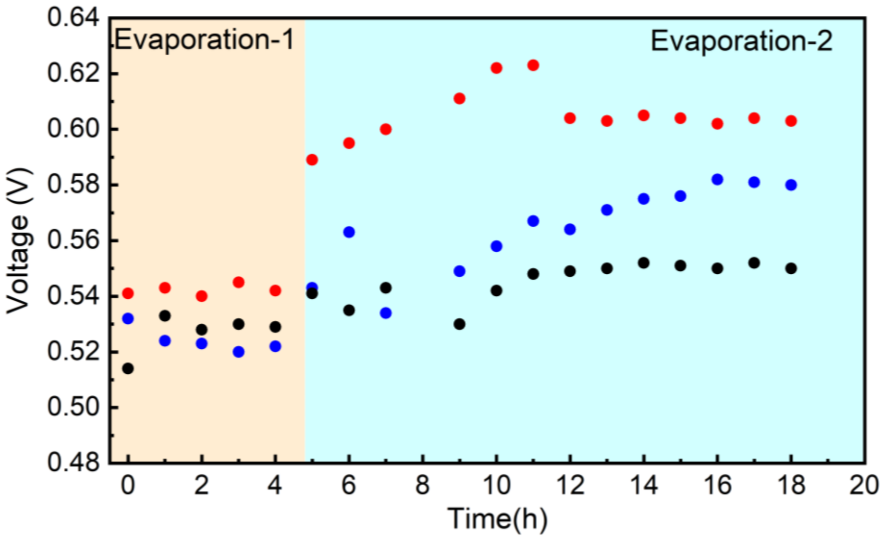

Figure S12. Stability and durability of voltage output at the optimal power generation performance at different evaporation process.
